# Downregulation of UBB potentiates SP1/VEGFA-dependent angiogenesis in clear cell renal cell carcinoma

**DOI:** 10.1038/s41388-024-03003-6

**Published:** 2024-03-11

**Authors:** Jinpeng Wang, Enyang Zhao, Bo Geng, Wei Zhang, Zhuolun Li, Qing Liu, Weiyang Liu, Wenfu Zhang, Wenbin Hou, Nan Zhang, Zhiming Liu, Bosen You, Pengfei Wu, Xuedong Li

**Affiliations:** 1 Department of Urology, The Second Affiliated Hospital of Harbin Medical University, Harbin, 150086 China; 2 Future Medical Laboratory, The Second Affiliated Hospital of Harbin Medical University, Harbin, 150086 China; 3 Department of Radiation Oncology, Urology, and Pathology, The Second Affiliated Hospital of Harbin Medical University, Harbin, 150086 China; 4 Department of Urology, Shanghai Fengxian District Central Hospital, Shanghai, 200233 China; 5 Department of Neurosurgery, The First Affiliated Hospital of USTC, Division of Life Sciences and Medicine, University of Science and Technology of China, Hefei, Anhui 230001 China; 6 Anhui Province Key Laboratory of Brain Function and Brain Disease, Hefei, Anhui 230001 China; 7 Anhui Provincial Stereotactic Neurosurgical Institute, Hefei, Anhui 230001 China; 8 Anhui Provincial Clinical Research Center for Neurosurgical Disease, Hefei, Anhui 230001 China; 9 Anhui Province Key Laboratory of Cancer Translational Medicine, Bengbu Medical University, 2600 Donghai Avenue, Bengbu, Anhui, 233030 China

**Keywords:** Tumour angiogenesis, Tumour biomarkers

## Abstract

Clear cell renal cell carcinoma (ccRCC) presents a unique profile characterized by high levels of angiogenesis and robust vascularization. Understanding the underlying mechanisms driving this heterogeneity is essential for developing effective therapeutic strategies. This study revealed that ubiquitin B (UBB) is downregulated in ccRCC, which adversely affects the survival of ccRCC patients. UBB exerts regulatory control over vascular endothelial growth factor A (VEGFA) by directly interacting with specificity protein 1 (SP1), consequently exerting significant influence on angiogenic processes. Subsequently, we validated that DNA methyltransferase 3 alpha (DNMT3A) is located in the promoter of UBB to epigenetically inhibit UBB transcription. Additionally, we found that an unharmonious UBB/VEGFA ratio mediates pazopanib resistance in ccRCC. These findings underscore the critical involvement of UBB in antiangiogenic therapy and unveil a novel therapeutic strategy for ccRCC.

## Introduction

Clear cell renal cell carcinoma (ccRCC) is one of the most common types of renal cell carcinomas and exhibits aggressive histology [1–3]. Our previous studies showed that the tumor microenvironment is an essential part of ccRCC, revealing 76, 105, and 48 genes associated with the ccRCC microenvironment in the TCGA-kidney renal clear cell carcinoma (TCGA-KIRC), GSE53000, and GSE53757 databases, respectively [4]. From these genes, we screened the tumor microenvironment-related gene UBB.

The expression of four specific genes in the human genome-RPS27A, UBA52, UBB, and UBC-exerts a crucial role in determining the levels of cellular ubiquitin [5]. UBB represents a protein-encoding gene implicated in the pathogenesis and progression of multiple disorders [6–9]. In ~30% of high-grade serous ovarian cancer patients, UBB is downregulated, and it presents a genetic abnormality in uterine carcinosarcoma and endometrial carcinoma [5]. In this study, our investigations demonstrated a correlation between the impaired expression of UBB and an unfavorable prognosis among ccRCC patients. Moreover, we elucidated that the diminished expression of UBB exerts a significant influence on both the angiogenesis ability and receptor tyrosine kinase inhibitor (TKI) resistance of RCC cells, achieved through direct interaction with SP1, a pivotal transcription factor regulating VEGFA.

CcRCC commonly presents with heterogeneous histological features, such as cytoplasm enriched with glycogen, eosinophilic cytoplasm, a fine vascular network, and enlarged thick vascular structures [10]. This heterogeneity is primarily attributed to mutations in von Hippel-Lindau (VHL), which result in the constitutive activation of hypoxia-inducible factors in ccRCC cells [11]. As a consequence, ccRCC cells display a pseudohypoxic phenotype characterized by the activation of vascular endothelial growth factor (VEGF), leading to their distinctive appearance [12]. Given the extensive neovascularization observed in more advanced stages of ccRCC, the therapeutic approach has predominantly revolved around targeting the VEGF signaling pathway using TKIs or monoclonal antibodies that specifically inhibit VEGF [13, 14]. Numerous TKIs currently serve as the predominant first-line treatment option for metastatic tumors, and they exhibit significant efficacy with respect to prolonging progression-free survival [11]. Nonetheless, a subset of patients displays inherent resistance, and the majority eventually acquire resistance, leading to an ultimate outcome of mortality [15]. Consequently, a pharmacologically superior and highly selective antiangiogenic therapy option is greatly needed as a viable strategy to optimize therapeutic efficacy. However, whether UBB regulates angiogenic processes by regulating the SP1-VEGFA axis in the ccRCC microenvironment remains poorly understood.

Previous studies have shown that UBB expression is highly significantly correlated with its methylation status in gynecological cancers [5]. Epigenetic mechanisms, including DNA methylation, exert pivotal control over gene expression, exhibiting profound influence not only in neoplastic cells but also across diverse immune cell populations [16]. Multiple investigations have consistently demonstrated that the methylation patterns of specific gene promoters can induce changes in cellular phenotypes and remodel the tumor microenvironment [17, 18]. Aberrant DNA methylation is widely acknowledged as a pivotal epigenetic hallmark in cancer, exerting a substantial influence on the transcriptional silencing of tumor suppressor genes [19]. DNA methylation is an epigenetic alteration facilitated by a group of enzymes known as DNA methyltransferases (DNMTs), comprising DNMT1, DNMT3A, DNMT3B, and DNMT3L [20]. This modification predominantly takes place within the CpG islands situated in the promoter region of genes, leading to the establishment of durable and heritable gene silencing mechanisms [21]. Recently, a multitude of studies have revealed that aberrant DNMT3A regulation contributes to the pathogenesis of various malignancies, including gastric cancer, acute erythroid leukemia, and oral cancer [22–24]. Our study provides evidence for the role of DNMT3A in facilitating the epigenetic silencing of UBB.

In summary, our study provides significant insights into the role of UBB in antiangiogenic therapy. UBB overexpression considerably suppressed RCC cells proliferation, tumor burden, and angiogenesis both in vitro and in vivo. We further demonstrated that UBB inhibited ccRCC angiogenesis by transcriptionally modulating VEGFA in an SP1-dependent manner. Our findings demonstrate the critical involvement of UBB in antiangiogenic therapy and also reveal that UBB holds substantial promise as a target for therapeutic intervention in ccRCC.

## Results

### UBB is downregulated in ccRCC and adversely impacts the survival of ccRCC patients

To explore the microenvironment-related genes within ccRCC, our previous studies performed ssGSEA and applied the ESTIMATE algorithm to generate corresponding scores in the ccRCC samples analyzed [4]. There were 76, 105, and 48 genes associated with the ccRCC microenvironment in the TCGA-KIRC, GSE53000, and GSE53757 datasets, respectively (Supplementary Fig. 1A). We updated 214 microenvironment-related genes in the STRING database to construct a protein-protein interaction network. Subsequently, the network was imported into Cytoscape to create subnetworks, revealing the top ten genes with at least ten interactions (Supplementary Fig. 1B). Among the ten genes examined, only UBB, HDAC1, MTOR, TLN1, and ACTN1 were found to hold prognostic significance using the univariate Cox proportional hazard model (Supplementary Fig. 1C). Kaplan-Meier survival analysis in the TCGA-KIRC database demonstrated that only high UBB (*p* < 0.001) and TLN1 (*p* = 0.025) mRNA expression was significantly associated with better overall survival compared to low expression (Supplementary Fig. 1D–H). However, TLN1 protein was found to be upregulated in tumor specimens (Supplementary Fig. 1I). Considering these results, further investigation will focus on UBB.

**Fig. 1:**
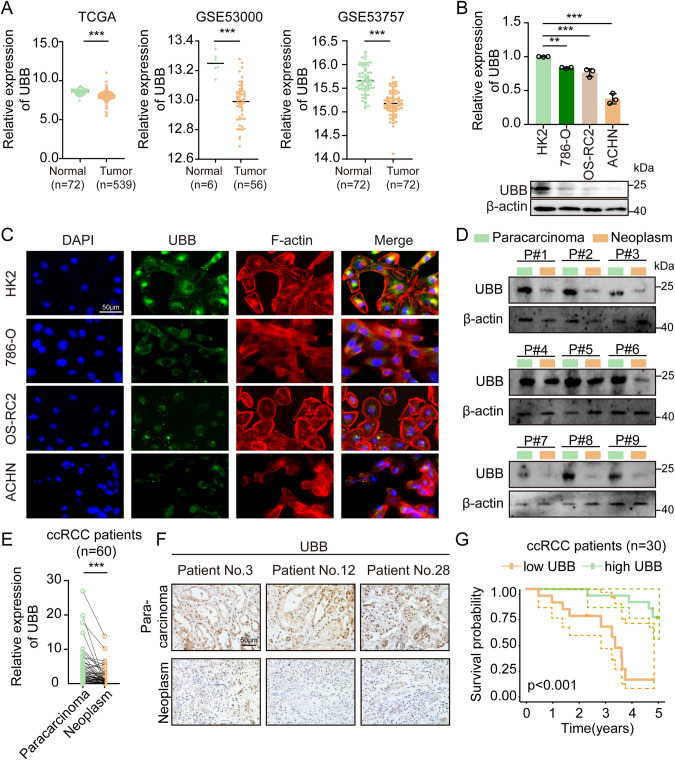
Diminished expression of UBB is observed in ccRCC and adversely impacts the survival of patients. **A** Analysis of UBB mRNA expression in TCGA-KIRC and GEO (GSE53000, GSE53757) datasets. **B** qPCR (top) and western blot (bottom) of UBB expression in the HK2 and RCC cell lines. **C** IF analysis of UBB expression in HK2 and RCC cell lines. Scale bar, 50 μm. **D** Western blot of UBB expression in 9 pairs of ccRCC and corresponding paracarcinoma specimens. **E** qPCR of UBB expression in 60 pairs of ccRCC and corresponding paracarcinoma specimens. **F** IHC of UBB expression in ccRCC and corresponding paracarcinoma specimens. The data are presented as a representative image. Scale bar, 50 μm. **G** Overall survival of 30 ccRCC patients according to Kaplan–Meier analysis. Data are presented as the mean ± SEM from three independent experiments. *p < 0.05, **p < 0.01, ***p < 0.001.

In TCGA-KIRC database, UBB levels showed a decreasing trend in tumor tissue compared to nontumor tissue, which was consistent with the results for the Gene Expression Omnibus (GEO) datasets (GSE53000, GSE53757) (Fig. 1A). Consistent with the data from the TCGA and GEO datasets, the expression of UBB at both the mRNA level and the protein level was substantially decreased in RCC cells (786-O, OS-RC2, and ACHN) compared to the normal proximal tubule epithelial cell line HK2 (Fig. 1B). Immunofluorescence staining results demonstrated that the expression of UBB was diminished in the RCC cells investigated (Fig. 1C). Furthermore, a pronounced reduction in UBB mRNA and protein levels was observed in ccRCC specimens vs. paired paracarcinoma specimens (Fig. 1D–F). Moreover, individuals diagnosed with ccRCC and relatively low UBB expression levels were observed to have a poorer prognosis than those with high expression levels (Fig. 1G). In summary, we identified a unique phenotype of UBB downregulation in ccRCC. The results above suggest that UBB could have a significant impact on the development and progression of ccRCC.

### UBB overexpression hindered tumor progression both in vitro and in vivo

**Fig. 2:**
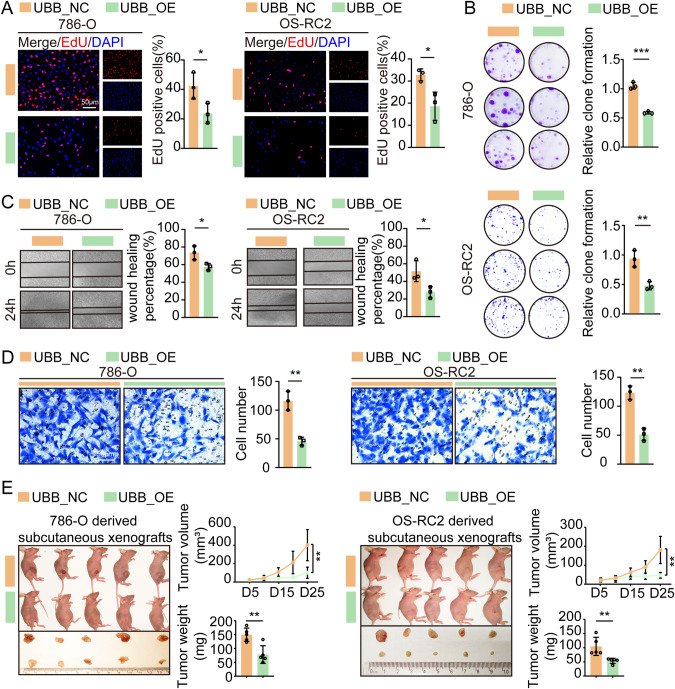
UBB hinders the proliferation and invasion of RCC cells and suppresses tumor growth in mice. **A** RCC cells proliferation after UBB overexpression was assessed by EdU assay. Scale bar, 50 μm. **B** RCC cells proliferation after UBB overexpression was assessed by clonogenic assays. **C** Wound healing assay of RCC cells after UBB overexpression. **D** Transwell assays were performed to evaluate invasion ability. Scale bar, 50 μm. **E** Subcutaneous xenografts (*n* = 5 per group) were excised from nude mice in the control group and UBB overexpression group. Tumor growth is represented by a line chart (top), and mean tumor weights are shown in the histogram (bottom). *p < 0.05, **p < 0.01, ***p < 0.001.

To investigate and validate the role of UBB in tumor progression in vitro, we conducted assays on cell viability, cell proliferation, cell migration, and cell invasion using RCC cells transfected with UBB overexpression lentivirus (UBB_OE). The results of the EdU assays and colony formation assays revealed a significant reduction in the viability and proliferation of RCC cells following UBB overexpression (Fig. 2A, B, Supplementary Fig. 2A, B). Furthermore, we observed that UBB overexpression suppressed the migration and invasion of RCC cells, as evidenced by wound healing and Transwell assays, in comparison to that of cells treated with negative control lentivirus (UBB_NC) (Fig. 2C, D, Supplementary Fig. 2C, D).

To validate the efficacy of UBB in vivo, nude mouse xenograft models were established. The growth of tumors was significantly inhibited following UBB overexpression (Fig. 2E). The tumors were weighed and sectioned for pathological analysis. The expression levels of Ki67 and CD31 were markedly decreased in the UBB_OE group compared to the UBB_NC group (Supplementary Fig. 2E). Hence, UBB serves as a tumor growth suppressor gene in ccRCC.

### Aberrant expression of UBB affects the capability of RCC cells to induce angiogenesis

**Fig. 3:**
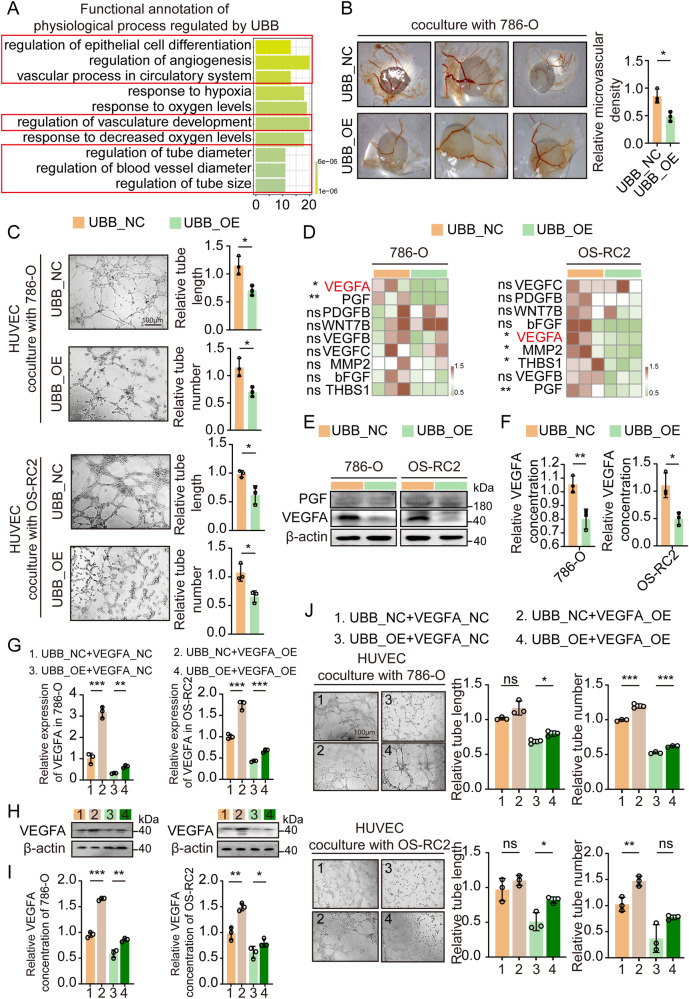
UBB suppressed the angiogenesis of RCC cells in vitro. **A** GO biological processes analysis. **B** Angiogenesis activity after UBB overexpression was assessed by CAM assay. **C** Representative capillary tubule structures were observed in HUVEC treated with culture medium from the indicated RCC cells (UBB_NC/UBB_OE). Scale bar, 100 μm. **D** qPCR analysis of cytokines and growth factors in the process of tumor angiogenesis. **E** Western blot analysis of VEGFA and PGF in RCC cells (UBB_NC/UBB_OE). **F** ELISA analysis of VEGFA culture medium from the indicated RCC cells (UBB_NC/UBB_OE). qPCR (**G**), western blot (**H**), and ELISA (**I**) of VEGFA from the indicated RCC cells (UBB_NC/UBB_OE and VEGFA_NC/VEGFA_OE). **J** Representative capillary tubule structures were observed in HUVEC treated with culture medium from the indicated RCC cells (UBB_NC/UBB_OE and VEGFA_NC/VEGFA_OE). Scale bar, 100 μm. Data are presented as the mean ± SEM from three independent experiments. *p < 0.05, **p < 0.01, ***p < 0.001.

Through a comprehensive analysis of the physiological process, we unveiled a potential association between low UBB expression and the activation of angiogenesis (Fig. 3A). Extensive literature indicates that angiogenesis is abnormally activated during the initiation and progression of ccRCC [14, 25–27]. By subjecting the specimen to histological staining, we uncovered a substantial augmentation in microvascular density within ccRCC (Supplementary Fig. 3A, B). Inducing overexpression of UBB prominently impeded the formation of vessels in the CAM assay and decreased the tube formation capability of HUVEC (Fig. 3B, C, Supplementary Fig. 3C).

Considering the multifaceted functions of various cytokines and growth factors in the process of tumor angiogenesis, we aimed to investigate whether UBB exerts its influence on angiogenesis by regulating the expression of key cytokines associated with angiogenesis [28]. Our study demonstrates that among the three analyzed RCC cells, VEGFA was the only cytokine that exhibited markedly suppressed levels of both mRNA and protein (Fig. 3D, E, Supplementary Fig. 3D). Utilizing the ELISA, it was observed that the overexpression of UBB resulted in a noteworthy decline in the concentration of VEGFA in the supernatants (Fig. 3F, Supplementary Fig. 3E). To investigate whether UBB’s tumor suppressor function in ccRCC relies on regulating VEGFA, we conducted concurrent overexpression of VEGFA to counteract its suppression caused by increased UBB levels in RCC cells. Consistent with the restoration of VEGFA mRNA, protein, and serum levels, the inhibition of HUVEC tube formation by UBB overexpression was alleviated following VEGFA overexpression (Fig. 3G–J, Supplementary Fig. 3F, G). Furthermore, we observed that VEGFA exhibited a close correlation with the prognosis of ccRCC and a higher expression level in neoplastic specimens than in paired paracarcinoma specimens (Supplementary Fig. 4A–E). Collectively, our findings provide evidence supporting the notion that UBB regulates angiogenesis by inducing the modulation of VEGFA expression.

### UBB modulates VEGFA expression via a direct physical interaction with SP1

**Fig. 4:**
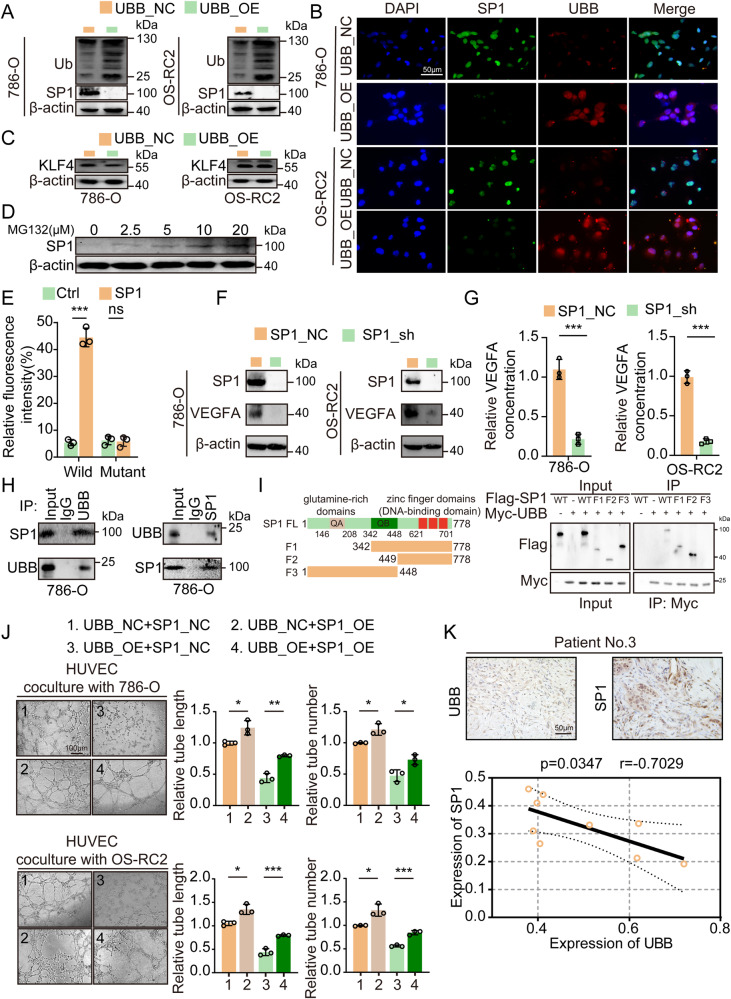
UBB interacts with SP1 to promote VEGFA transcription. **A** Western blot analysis of the polyubiquitination level and expression level of SP1 in RCC cells (UBB_NC/UBB_OE). **B** IF analysis of UBB and SP1 in RCC cells (UBB_NC/UBB_OE). Scale bar, 50 μm. **C** Western blot analysis of KLF4 in RCC cells (UBB_NC/UBB_OE). **D** Western blot analysis of SP1 expression in MG132-treated RCC cells (786-O UBB_OE). **E** VEGFA promoter luciferase reporter plasmids and Renilla pRL-TK plasmids were transfected into empty vector-transduced and SP1-transduced RCC cells. Forty-eight hours later, the luciferase signal was examined. **F** Western blot analysis of SP1 and VEGFA expression in RCC cells (SP1_NC/SP1_sh). **G** ELISA analysis of VEGFA in culture medium from the indicated RCC cells (SP1_NC/SP1_sh). **H** Co-IP assay showing that endogenous UBB interacted with endogenous SP1 in 786-O cells. **I** Flag-tagged SP1 (full length or truncations) and Myc-tagged UBB were transfected into HEK293T cells. Co-IP assays revealed that UBB interacted with FL, F1, and F2 but not with F3. **J** Representative capillary tubule structures were observed in HUVEC treated with culture medium from the indicated RCC cells (UBB_NC/UBB_OE and SP1_NC/SP1_OE). Scale bar, 100 μm. **K** Representative results (top) and correlation (bottom) between UBB expression and SP1 expression. Scale bar, 50 μm. Data are presented as the mean ± SEM from three independent experiments. *p < 0.05, **p < 0.01, ***p < 0.001.

With the aim of delving deeper into the mechanistic insights behind UBB-mediated downregulation of VEGFA expression, we employed the TRRUST and JASPAR databases for an analysis of VEGFA transcription factors (Supplementary Fig. 5A). Interestingly, we observed that SP1 and KLF4 exhibit a shared transcriptional orientation with VEGFA, and only SP1 (not KLF4) expression was noticeably inhibited upon UBB overexpression and increased levels of ubiquitination (Fig. 4A–C, Supplementary Fig. 5B–D). RCC cells exhibiting UBB overexpression were subjected to treatment with MG132, resulting in significant upregulation of SP1 (Fig. 4D). SP1, an important transcription factor, is involved in the progression of various cancers [29–31]. In ccRCC, the expression of SP1 was consistently increased at the mRNA and protein levels (Supplementary Fig. 5E–G). A luciferase experiment revealed that the expression of the reporter gene linked to the wild-type VEGFA construct was significantly activated upon treatment with SP1 overexpression, while the mutant-type VEGFA constructs completely abolished the activation effect of SP1 (Fig. 4E). Silencing of SP1 resulted in downregulation of VEGFA, accompanied by diminished extracellular VEGFA secretion and HUVEC tube formation (Fig. 4F, G, Supplementary Fig. 5H).

Co-IP analyses revealed the physical interaction between UBB and SP1 in cells (Fig. 4H). We also revealed that UBB specifically interacts with SP1-FL, SP1-F1, and SP1-F2, indicating the requirement of amino acids 449–778 for UBB-SP1 interaction (Fig. 4I). In light of the observed downregulation of SP1 protein expression induced by UBB in ccRCC, our research endeavored to elucidate the impact of UBB on angiogenesis in HUVEC through an SP1-dependent mechanism. Significantly, our study revealed that the suppression of HUVEC tube formation caused by UBB was mitigated upon the introduction of SP1 (Fig. 4J). IHC analysis revealed a negative correlation between UBB and SP1 expression in ccRCC specimens (Fig. 4K, Supplementary Fig. 5I). These results support the essential role of SP1 in facilitating the UBB-mediated upregulation of VEGFA.

### Epigenetic silencing facilitated by DNMT3A contributes to the suppressed transcription of UBB in ccRCC

**Fig. 5:**
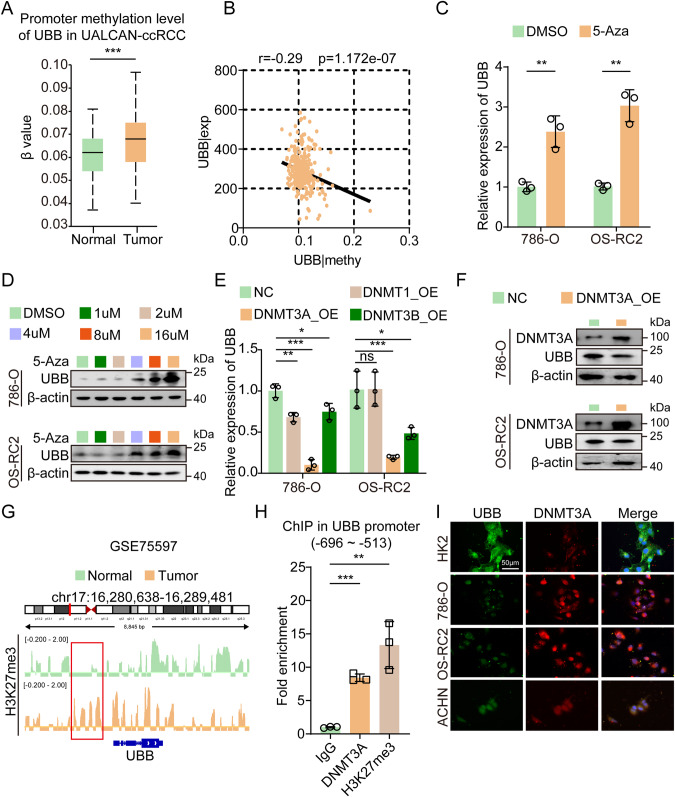
Epigenetic silencing maintained by DNMT3A contributes to suppressed transcription of UBB in ccRCC. **A** Promoter methylation level analysis of UBB in ccRCC based on data from the UALCAN-ccRCC dataset. **B** Correlation between UBB expression level and promoter methylation level based on data from the TCGA-KIRC database. qPCR (**C**) and western blot (**D**) analysis of UBB expression in RCC cells treated with 5-Aza. **E** qPCR analysis of UBB mRNA levels in RCC cells transfected with NC, DNMT1_OE, DNMT3A_OE, or DNMT3B_OE. **F** Western blot of DNMT3A and UBB expression in RCC cells transfected with NC and DNMT3A_OE. **G** The IGV browser image of H3K27me3 enrichment in the UBB promoter region in ccRCC. **H** ChIP-qPCR analysis of DNMT3A genomic occupancy and H3K27me3 methylation status at the UBB promoter. **I** IF analysis of DNMT3A and UBB expression in HK2 and RCC cell lines. Scale bar, 50 μm. Data are presented as the mean ± SEM from three independent experiments. *p < 0.05, **p < 0.01, ***p < 0.001.

Within the domain of genomic research, comprehensive examination has revealed that alterations in the methylation profiles of gene promoters underlie the aberrant expression of multiple genes [20]. Considering the substantial abundance of CpG islands within the UBB promoter region, our conjecture postulated that epigenetic modifications had a pivotal impact on the attenuation of UBB expression. To investigate the potential inhibitory effect of methylation on UBB expression, we conducted an analysis of the methylation status within the promoter region of UBB and the correlation between UBB expression levels and methylation levels. The data indicated a significant methylation pattern within the UBB promoter region in ccRCC, showing an inverse correlation with UBB expression levels (Fig. 5A, B). After treating RCC cells with 2′-deoxy-5-azacytidine (5-Aza), a potent inhibitor of DNMTs, we observed a remarkable augmentation of UBB expression (Fig. 5C, D). As a cytidine nucleoside analog, 5-Aza selectively hinders DNA methylation by impairing its catalytic activity [32]. Our investigations involving the manipulation of DNMTs expression in RCC cells specifically demonstrated that only DNMT3A exerts notable regulatory control exclusively over the expression of UBB (Fig. 5E, F). DNMT3A is involved in the process of trimethylating Lys-27 in histone 3 (H3K27me3) and DNA methylation [33]. In ccRCC, the Integrative Genomics Viewer (IGV) plots revealed higher enrichment of H3K27me3 in the UBB promoter region, indicating its dominant role in regulating UBB expression (Fig. 5G). To determine the precise localization of DNMT3A within the UBB promoter, we employed a ChIP assay, which revealed a notable enrichment of DNMT3A specifically within the genomic region spanning nucleotides −696 to −513 (Fig. 5H). Additionally, we observed a similar enrichment pattern for H3K27me3 in this particular region (Fig. 5H). In particular, we observed notable upregulation of DNMT3A in ccRCC (Supplementary Fig. 6A–C). In addition, our study uncovered a significant negative correlation between the expression of DNMT3A and UBB (Fig. 5I, Supplementary Fig. 6D). Our findings unequivocally support the proposition that the epigenetic suppression of UBB in ccRCC involves the actions of DNMT3A.

### Unharmonious UBB/VEGFA ratio mediates pazopanib resistance in ccRCC

**Fig. 6:**
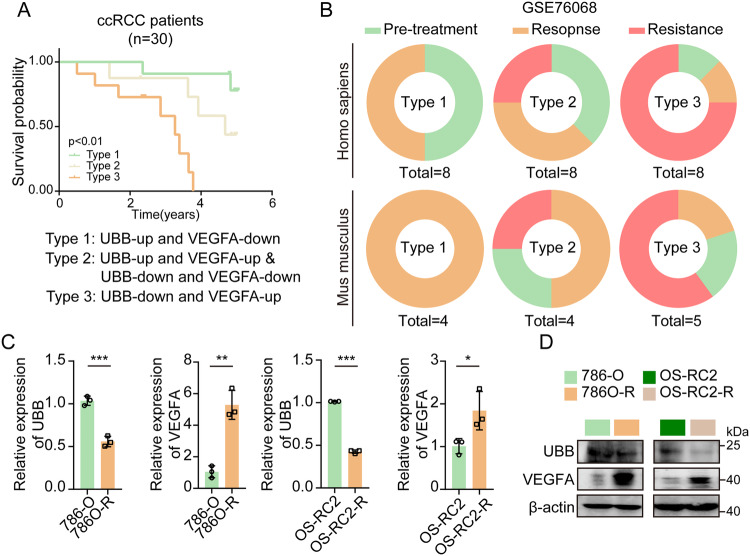
UBB/VEGFA ratio affects ccRCC prognosis and pazopanib resistance. **A** Kaplan-Meier survival analysis showed the correlation of the UBB/VEGFA ratio with the survival rate of ccRCC patients. **B** The correlation of risk group with TKI resistance in ccRCC. qPCR (**C**) and western blot (**D**) analysis of UBB and VEGFA expression in TKI-resistant RCC cells. Data are presented as the mean ± SEM from three independent experiments. *p < 0.05, **p < 0.01, ***p < 0.001.

We devised a criterion to investigate the correlation between the ratio of UBB/VEGFA and the progression of ccRCC, which involved identifying the expression of UBB and VEGFA in ccRCC tissues. Subsequently, we meticulously categorized ccRCC patients into three distinct groups based on UBB and VEGFA expression (Types 1, 2, and 3). Investigation using Kaplan-Meier survival analysis revealed a significant association between the concurrent downregulation of UBB and upregulation of VEGFA expression in ccRCC patients and poorer overall survival outcomes (Type 3) (Fig. 6A). Moreover, it was evident that the Type 3 group exhibited a notably higher proportion of TKI-resistant patients and TKI-resistant patient-derived xenograft (PDX) models than the other two groups, indicating that ccRCC patients with reduced UBB levels coinciding with elevated VEGFA levels illustrated a propensity for TKI resistance (Fig. 6B).

Consequently, to further examine the manifestation of UBB and VEGFA expression, we engineered pazopanib-resistant cell lines. RCC cells (786-O, OS-RC2) were exposed to gradually increasing concentrations of pazopanib and underwent repetitive cycles of pazopanib treatment for a duration of eight months. As a result, RCC cells developed a resistant phenotype toward pazopanib, and these cell lines were named 786-O-R and OS-RC2-R. The activation level of UBB was significantly inhibited, while VEGFA was dramatically upregulated in pazopanib-resistant cells, suggesting that UBB and VEGFA might act as key genes to reverse pazopanib resistance (Fig. 6C, D). Taken together, these results reveal that the UBB/VEGFA ratio impacts ccRCC prognosis and resistance to pazopanib.

## Discussion

CcRCC represents the predominant subtype of RCC and is characterized by enhanced hypoxia and upregulation of angiogenesis-associated genes due to early VHL inactivation [34]. Based on ccRCC clinical samples, we found substantial augmentation in microvascular density within tumor tissue. Microvascular density is commonly utilized as an indicator of tumor angiogenesis, and in the case of most solid tumors, an elevated microvascular density is associated with a more unfavorable prognosis [35]. Dysregulation of angiogenesis is implicated in the progression of ccRCC [36]. The tumor microenvironment exerts a significant influence on the regulation of angiogenesis within the tumor [28]. Extrinsic regulation of angiogenesis by the tumor microenvironment, highlighting potential vulnerabilities that could be targeted to improve the applicability and reach of anti-angiogenic cancer therapies.

Our study observed that diminished microenvironment-related UBB expression correlated with poorer prognosis in patients. The in vitro assay and in vivo tumor growth assay revealed that UBB overexpression markedly suppressed tumor growth and intratumoral vascularization. Physiological process analysis revealed a potential association between reduced UBB expression and enhanced angiogenic activation. Considering that UBB regulated VEGFA expression at both mRNA and protein levels, we further identified the transcriptional regulons of VEGFA. From candidate transcription factors, we identified SP1 as a positive regulator of VEGFA and had a direct physical interaction with UBB. These results indicated that UBB regulated the expression of VEGFA in a SP1-dependent manner, thereby modulating the angiogenic capability of RCC cells.

The VEGF signaling pathway serves as the principal mediator of angiogenesis and plays a crucial role in promoting tumor initiation and progression [25]. VEGF signaling is integral to the maintenance of vascular homeostasis [37]. The VEGF family of growth factors is composed of five members in mammals and they interact with three transmembrane tyrosine kinase receptors [38]. TKIs, such as pazopanib, exert their therapeutic effects by suppressing the VEGF signaling pathway and inhibiting tumor angiogenesis [39]. Consequently, TKIs have emerged as the first-line treatment option for advanced ccRCC [39]. Notwithstanding the transformative impact of targeted therapies on the therapeutic paradigm for advanced ccRCC following the cytokine era, the emergence of acquired TKI resistance has emerged as a formidable challenge afflicting a substantial cohort of ccRCC patients. Due to the dearth of reliable predictive and diagnostic biomarkers for TKI resistance, our study aimed to identify molecular markers intricately linked to the outcomes of ccRCC patients undergoing treatment with TKIs, with the ultimate goal of facilitating the development of personalized treatment approaches for ccRCC.

In this study, the primary objective was to provide precise clinical guidance for the administration of TKIs to ccRCC patients, leveraging the potential of discriminating molecular indicators. We employed a meticulous approach to categorize ccRCC into three types according to UBB and VEGFA expression patterns (Fig. 6A). Our study elucidated that Type 3 patients demonstrated the most unfavorable prognosis, characterized by a concomitant decrease in UBB expression and an increase in VEGFA expression. Furthermore, our research findings suggest a heightened susceptibility to drug resistance in patients receiving TKI treatment classified as Type 3, and the results were consistent for the corresponding PDX model. This preliminary investigation exemplifies the prospective significance of UBB and VEGFA expression levels in predicting the effectiveness of TKI treatment in ccRCC, suggesting the reliability of using the UBB/VEGFA ratio as an indicator in future clinical applications.

In a study involving a substantial cohort of ccRCC samples, TCGA analysis revealed a significant association between abnormal hypermethylation and both the stage and grade of ccRCC [40]. Given the considerable prevalence of CpG islands within the UBB promoter region, our research focus shifted toward exploring the epigenetic modulation of UBB. The expression of UBB was elevated following treatment with 5-Aza, thereby suggesting a potential influence of 5-Aza on the upregulation of UBB expression. Additionally, our findings suggest that DNMT3A plays a pronounced role in repressing the transcriptional activity of UBB. This hypothesis is further supported by the data presented in Fig. 5F, which showed substantial inhibition of UBB expression upon specific overexpression of DNMT3A.

During this investigation, we uncovered UBB as a critical regulator of ccRCC angiogenesis. We sought to determine if UBB could be drugged to take advantage of its anti-angiogenic function in the next research. Patients receiving cancer treatment commonly integrate complementary and alternative medicine into their healthcare protocol, encompassing the use of herbs and supplements. Studies have indicated that flavonoids exhibit effects on both antiangiogenic and in vivo anti-metastatic models [41]. Flavonoids primarily include glycitein, genistein, and daidzein [41–43]. In the next phase of our research, we plan to verify whether glycitein can enhance the activity of UBB to counteract tumor vasculature and synergize with pazopanib, thereby increasing its sensitivity, thus developing potential therapeutic agents against pazopanib resistance.

**Fig. 7:**
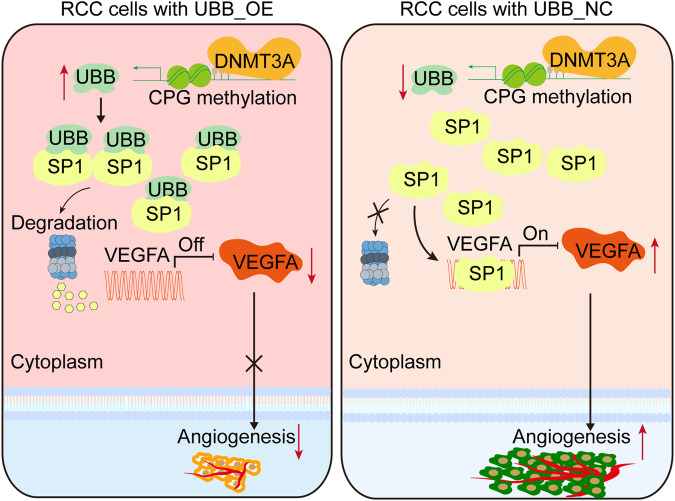
The mechanistic scheme of UBB in ccRCC. Model showing the antitumor mechanism by which UBB affects angiogenesis via the SP1-VEGFA signaling pathway.

In summary, our study reveals the importance of UBB in retarding ccRCC growth and angiogenesis via SP1/VEGFA signaling (Fig. 7). The potential of UBB in enhancing antiangiogenic therapy and overcoming pazopanib resistance represents a promising and innovative approach for mitigating ccRCC. This prospective therapeutic strategy warrants further evaluation in subsequent studies to ascertain its effectiveness and feasibility.

## Materials and methods

### Patients and samples

From April 1, 2018 to August 1, 2023, a total of 60 ccRCC tissue samples, confirmed through histological examination, were acquired from the Department of Urology at the Second Affiliated Hospital of Harbin Medical University in Harbin, China. Additionally, 60 paired adjacent noncancerous tissues were obtained. The researchers obtained prior approval from the hospital’s institutional review board and conducted in accordance with the principles expressed in the Declaration at Helsinki. Written informed consent was received from all participants. Supplementary Table 1 presents information on patients with follow-up data.

### Statistical analysis

Statistical analysis was performed using various methods. Student’s *t* test was used to evaluate differences between variable groups. For comparisons involving at least three groups, one-way analysis of variance was employed. Univariate survival analyses were conducted using Kaplan-Meier curves and log-rank tests. Figures were generated using R version 4.3.0 with the pheatmap extension package. Circos software and IGV were used for ChIP-seq data visualization. Statistical significance was determined by a *p* value less than 0.05. All statistical analyses were performed using GraphPad software version 7.0 (GraphPad Software, CA, USA).

More materials and methods are included in the Supplementary files due to the space limit (Supplementary Materials and Methods).

### Supplementary information

Supplementary Figure legends

Supplementary Materials & Methods

Supplementary Figure 1

Supplementary Figure 2

Supplementary Figure 3

Supplementary Figure 4

Supplementary Figure 5

Supplementary Figure 6

Supplementary table 1

Supplementary Table 2

Supplementary Table 3

Supplementary Table 4

## Data Availability

All data in our study are available upon request.
