# Supplementary material for: Downregulation of UBB potentiates SP1/VEGFA-dependent angiogenesis in clear cell renal cell carcinoma
Source: Oncogene. 2024 Mar 11;43(18):1386–96. doi: 10.1038/s41388-024-03003-6 (PMC11065696; doi:10.1038/s41388-024-03003-6)
Supplement: Supplementary file 1 — Supplementary Figure legends [file 41388_2024_3003_MOESM1_ESM.docx]

**Supplementary Figure legends**

Supplementary Figure 1. Screening the tumor microenvironment-related gene UBB in ccRCC. A. The number of microenvironment-related genes in the TCGA, GSE53000, and GSE53757 datasets. B. PPI interaction network of 214 microenvironmental genes. C. Forest plot showing the univariate Cox proportional hazard model. Overall survival of UBB (D), HDAC1 (E), MTOR (F), TLN1 (G), and ACTN1 (H) in the TCGA-KIRC cohort according to Kaplan-Meier analysis. I. Relative protein expression of TLN1 in the UALCAN-ccRCC dataset.

Supplementary Figure 2. UBB suppresses the proliferation and metastasis of ACHN cells. A. ACHN cells proliferation after UBB overexpression was assessed by EdU assay. Scale bar, 50 μm. B. ACHN cells proliferation after UBB overexpression was assessed by clonogenic assays. C. Wound healing assay of ACHN cells after UBB overexpression. D. Transwell assays were performed to evaluate invasion ability. Scale bar, 50 μm. Data are presented as the mean ± SEM from three independent experiments. E. Representative H&E staining, IHC of Ki67, and IF of UBB and CD31. Scale bar, 100 μm. Data are presented as the mean ± SEM from three independent experiments. *p < 0.05, **p < 0.01, ***p < 0.001.

Supplementary Figure 3. UBB suppressed the angiogenesis of ACHN in vitro. A. Representative H&E staining of paracarcinoma and ccRCC specimens. Scale bar, 100 μm. B. Representative IHC of CD31 in paracarcinoma and neoplasm specimens. Scale bar, 50 μm. C. Representative capillary tubule structures were observed in HUVEC treated with culture medium from ACHN cells (UBB_NC/UBB_OE). Scale bar, 100 μm. D. qPCR analysis of cytokines and growth factors in the process of tumor angiogenesis (left panel). Western blot analysis of VEGFA and PGF in ACHN (UBB_NC/UBB_OE) (right panel). E. ELISA analysis of VEGFA culture medium from ACHN cells (UBB_NC/UBB_OE). F. qPCR (top), western blot (left), and ELISA (right) analysis of VEGFA from ACHN cells (UBB_NC/UBB_OE and VEGFA_NC/VEGFA_OE). G. Representative capillary tubule structures were observed in HUVEC treated with culture medium from ACHN cells (UBB_NC/UBB_OE and VEGFA_NC/VEGFA_OE). Scale bar, 100 μm. Data are presented as the mean ± SEM from three independent experiments. *p < 0.05, **p < 0.01, ***p < 0.001.

Supplementary Figure 4. Increasing expression of VEGFA is associated with poor prognosis in ccRCC. A. VEGFA expression level was correlated with tumor grade in UALCAN-ccRCC patient survival. B. Analysis of VEGFA expression in the TCGA-KIRC and CPTAC-ccRCC databases. C. qPCR of VEGFA expression in 30 pairs of ccRCC and corresponding paracarcinoma specimens. D. Western blot of VEGFA expression in 9 pairs of ccRCC and corresponding paracarcinoma specimens. E. IHC of VEGFA expression in ccRCC and corresponding paracarcinoma specimens. The data are presented as a representative image. Scale bar, 50 μm. Data are presented as the mean ± SEM from three independent experiments. *p < 0.05, **p < 0.01, ***p < 0.001.

Supplementary Figure 5. SP1 enhanced the angiogenesis of RCC cells in vitro. A. VEGFA transcription factors were screened using the TRUSST and JASPAR databases. B. Western blot analysis of the polyubiquitination level and expression level of SP1 in ACHN cells (UBB_NC/UBB_OE). C. Western blot analysis of KLF4 in ACHN cells (UBB_NC/UBB_OE). D. IF analysis of UBB and SP1 in ACHN cells (UBB_NC/UBB_OE). Scale bar, 50 μm. E. qPCR and western blot analysis of SP1 in RCC cells. F. Western blot of SP1 expression in 9 pairs of ccRCC and corresponding paracarcinoma specimens. G. IHC of SP1 expression in ccRCC and corresponding paracarcinoma specimens. The data are presented as a representative image. Scale bar, 50 μm. H. Representative capillary tubule structures were observed in HUVEC treated with culture medium from RCC cells (SP1_NC/SP1_sh). Scale bar, 100 μm. I. IHC of UBB and SP1 expression in paired ccRCC samples. Scale bar, 50 μm. Data are presented as the mean ± SEM from three independent experiments. *p < 0.05, **p < 0.01, ***p < 0.001.

Supplementary Figure 6. DNMT3A is upregulated in ccRCC. A. Expression levels of DNMT3A in the TCGA-KIRC and GEO database. B. IHC of DNMT3A expression in ccRCC and corresponding paracarcinoma specimens. The data are presented as a representative image. Scale bar, 50 μm. C. Western blot of DNMT3A expression in 9 pairs of ccRCC and corresponding paracarcinoma specimens. D. The correlation between UBB expression and DNMT3A expression in the TCGA-KIRC database. Data are presented as the mean ± SEM from three independent experiments. *p < 0.05, **p < 0.01, ***p < 0.001.
