# Supplementary material for: Downregulation of UBB potentiates SP1/VEGFA-dependent angiogenesis in clear cell renal cell carcinoma
Source: Oncogene. 2024 Mar 11;43(18):1386–96. doi: 10.1038/s41388-024-03003-6 (PMC11065696; doi:10.1038/s41388-024-03003-6)
Supplement: Supplementary file 2 — Supplementary Materials & Methods [file 41388_2024_3003_MOESM2_ESM.docx]

**Supplementary Materials & Methods**

**Cell culture**

The RCC cell lines 786-O, OS-RC2, and ACHN; the human immortalized proximal tubule epithelial cell line HK2; human umbilical vein endothelial cells HUVEC; and HEK293T cells were obtained from the American Type Culture Collection (ATCC, Manassas, VA). 786-O, OS-RC2, ACHN and HEK293T cells and HUVEC were cultured in 1640 medium, while HK2 cells were cultured in DMEM/F12 medium. Both types of media were supplemented with 10% fetal bovine serum (FBS) and antibiotics (100 units/ml penicillin, 100 mg/ml streptomycin). The cell lines were maintained at a temperature of 37°C in a humidified environment with 5% CO2.

**HUVEC tube formation assay**

HUVEC were used in the tube formation assay. Matrigel (BD Biosciences, CA, USA) was added to a 24-well plate (Jetbiofil, Guangzhou, China) and allowed to polymerize at 37°C for 30 minutes. Then, 2×10^4^ HUVEC in 200 µL of conditioned medium were added to each well and incubated at 37°C in 5% CO2 for 20 hours. Bright-field images were captured at 200× magnification using a microscope (Leica DM2500P, Germany). The degree of tube formation was compared to the formation of cell strings.

**Chicken chorioallantoic membrane (CAM) assay**

To assess the direct impact on angiogenesis, an 8th-day CAM assay was performed on fertilized chicken eggs (Yueqin Breeding Co. Ltd, Guangdong, China). An opening with a diameter of 1 cm was created in the shell of each egg containing an 8-day-old chicken embryo. The dermic sheet covering the floor of the air sac was carefully removed to expose the CAM. Filter paper with a diameter of 0.5 cm was placed on top of the CAM, and 100 μL of conditioned medium harvested from transduced glioma cells was added to the center of the paper. The window was then sealed with sterile adhesive tape, and the eggs were incubated at 37°C under 80-90% relative humidity for 48 hours. After fixation with a solution of methanol and acetone (1:1) for 15 minutes, the CAM was dissected and collected, and gross photographs were taken. The effect of the conditioned media was evaluated by comparing the number of second- and third-order vessels between this group and the group treated with medium harvested from the control group. Statistical analysis of number the second- and third-order vessels was performed using a two-tailed Student's t test.

**Western blot analysis**

Western blotting was carried out following the same procedure as previously detailed [1]. Protein samples (30-50 μg) were lysed in cell lysis buffer, separated on an SDS/PAGE gel, transferred onto PVDF membranes, and incubated with primary antibodies (Supplementary Table 2) overnight, followed by HRP-conjugated secondary antibodies (Zsbio Store-bio, Beijing, China) for 1 h. Visualization was performed using a ChemiDocTM MP Imaging System (Bio-Rad, CA, USA).

**Lentivirus packaging and transfection**

Lentivirus packaging and transfection were carried out following the same procedure as previously described [1]. Lentivirus particles were produced by cotransfecting pLKO.1 (for shRNA plasmids) and pWPI (for overexpression) vectors with psPAX2 and pMD2. G plasmids into HEK293T cells using CaCl2 transfection. After 48 hours, lentivirus particles were harvested, filtered, and stored at -80°C or used immediately. The human SP1 gene and its truncations were amplified from cDNA using PCR and cloned and inserted into the pSin-EF2-puro vector. The human SP1 gene and its truncations were amplified from cDNA using PCR and cloned into the pSin-EF2-puro vector. The nucleotide sequence in short hairpin RNA (shRNA) against SP1was: CCGGCCCAAGTTTATTTCTCTCTTACTCGAGTAAGAGAGAAATAAACTTGGGTTTTT.

**RNA extraction and quantitative real-time PCR (qPCR) analysis**

RNA extraction and qPCR analysis were carried out following the same procedure as previously detailed [2]. Total RNA was extracted using TRIzol reagent (Invitrogen, USA), reverse transcribed with the PrimeScript RT Reagent Kit (Takara, Japan), and quantified by qRT-PCR using SYBR green (Takara, Japan) on a Bio-Rad CFX96 system. The expression of UBB, VEGFA, VEGFB, VEGFC, PGF, PDGFB, WNT7B, MMP2, bFGF, THBS1, and SP1 was normalized to GAPDH (mRNA) using the 2^−ΔΔCt^ method with primers listed in Supplementary Table 3.

**Animal study and tumor treatment**

Nude mouse xenograft models were established using four- to five-week-old BALB/c nude female mice (Beijing Vital River, Beijing, China). The mice were randomly and blindly divided into groups (n = 5 mice/group). Single-cell suspensions of 3 × 10^6^ cells (786-O_UBB_NC, 786-O_UBB_OE, OS-RC2 _UBB_NC, OS-RC2 _UBB_OE) were injected subcutaneously into the dorsal flanks of the mice. Tumor measurements were taken twice a week, and the tumor volume was calculated using the following formula: total tumor volume (mm^3^) = π/6×L×W^2. After 4 or 5 weeks, the mice were humanely euthanized, and the tumors were excised for further experimentation. All mice were kept in a clean and controlled environment at the animal facility of Harbin Medical University, China. The animal experiments conducted were ethically approved and closely monitored by the Harbin Medical University Institutional Animal Use and Care Committee.

**Coimmunoprecipitation analysis (Co-IP)**

The protein in the cell lysates was immunoprecipitated using antibodies, including anti-Flag (Sigma-Aldrich, USA), anti-Myc (Sigma-Aldrich, USA), anti-UBB antibody (Proteintech, IL, USA), and anti-SP1 antibody (Proteintech, IL, USA). The resulting immunocomplexes were then analyzed using antibodies targeting Flag (Sigma-Aldrich), Myc (Sigma-Aldrich), UBB (Proteintech, IL, USA), and SP1 (Proteintech, IL, USA). Detection was achieved using an ECL detection system.

**Luciferase reporter assay**

Luciferase reporter assay was carried out following the same procedure as previously detailed [3]. To perform the luciferase reporter assay, cells were seeded in 96-well plates and transfected with the GV272 luciferase vector (GeneChem, Shanghai, China). Mutant and wild-type VEGFA constructs with potential SP1 binding sites were generated and fused to the GV272 luciferase reporter vector. Firefly luciferase activity was measured in each well using the Dual Luciferase Reporter Assay System (Promega, USA) and normalized to Renilla luciferase activity following the manufacturer's instructions.

**Enzyme-linked immunosorbent assay (ELISA)**

ELISA was conducted on conditioned media collected from each cell culture. ELISA kits were used to measure the levels of secreted VEGFA (Ruifan, Shanghai, China) following the manufacturer's instructions. The collected CM was filtered through a 0.22-μm filter and stored at −80°C.

**Immunofluorescence (IF)**

IF was performed to visualize protein expression and localization in cells. Briefly, cells were seeded on cell slides (WHB-24-CS, Shanghai, China) in 24-well tissue culture plates and fixed using standard procedures after overnight incubation at 4°C. Primary antibodies (UBB, VEGFA, SP1, CD31, and DNMT3A) were diluted in 1% BSA and incubated with the cells overnight at 4°C. The cells were washed three times with PBS and then incubated with secondary antibody (Alexa Fluor 488 and 594, Thermo Fisher) for 1 h at room temperature. The nuclei were stained with DAPI (Sigma, USA), and images were captured using a fluorescence microscope (Nikon C2, Tokyo, Japan).

**Colony formation assay**

Cells were plated at a density of 0.1 × 10³ cells per well in a six-well plate (Jetbiofil, Guangzhou, China) and incubated for 14 days. After washing twice with PBS, the colonies were fixed with 4% paraformaldehyde for 15 minutes and stained with 0.1% crystal violet for 30 minutes. The number of colonies was captured using a ChemiDocTM MP Imaging System (Bio-Rad, CA, USA) and quantified using ImageJ software.

**EdU assay**

RCC cells were seeded on tissue-culture slides, treated and incubated with 10 µM EdU for 24 h (Ribobio, Guangzhou, China) according to the manufacturer’s instructions. The proportion of cells that incorporated EdU was determined with a fluorescence microscope.

**Transwell migration assay**

In a Transwell migration assay, a total of 2×10^4^ RCC cells were plated on the upper side of a polycarbonate Transwell filter (Jetbiofil, Guangzhou, China). The RCC cells were then incubated at 37°C for 24 hours. After the incubation period, the RCC cells remaining on the upper surface were removed using cotton swabs. The RCC cells that had migrated to the lower membrane surface were fixed with 1% paraformaldehyde, stained with crystal violet, and counted under an optical microscope at ×400 magnification.

**Wound healing assay**

RCC cells were seeded on a six-well plate to form a confluent monolayer in 10% FBS-containing medium. The monolayer was scratched, washed, and incubated with 0.5% FBS-containing RPMI1640 for 24 hours. Wound closure was measured in three random fields at 200× magnification using ImageJ software. The rate of wound healing was calculated as the migrated cell surface area/total surface area × 100.

**Hematoxylin and eosin (HE) staining and immunohistochemistry (IHC)**

Tissues were subjected to HE staining and IHC. IHC was performed on 5 μm paraffin sections using a three-step process and a DAB staining kit (ZSGB-BIO, Beijing, China). Formalin-fixed, paraffin-embedded tissue sections were first dewaxed in xylene, rinsed in graded ethanol, and rehydrated in double-distilled water. The slides were pretreated by steaming them in sodium citrate buffer for 15 minutes at 95°C to retrieve antigens. Primary antibodies against UBB, SP1, VEGFA, CD31, and DNMT3A were applied and incubated at 4°C overnight. Following washing in PBS buffer, the tissues were covered with an anti-mouse/rabbit polymer HRP-label (ZSGB-BIO, Beijing, China) for 30 minutes. DAB chromogen solution was added, and the samples were incubated for approximately 1 minute to allow proper brown color development.

**Chromatin immunoprecipitation (ChIP)**

First, cell lysates were subjected to precleaning using protein A-agarose-conjugated normal IgG (sc-2027, Santa Cruz). Subsequently, anti-DNMT3A antibody (Cell Signaling Technology) and anti-H3K23me3 antibody (Abcam) were added to the cell lysates and incubated overnight at 4°C, while IgG served as the negative control. For qPCR analysis, specific primer sets designed to amplify the target sequence within the UBB promoter were utilized (primers listed in Supplementary Table 4).

**References**

1 Liu Q, Zhao E, Geng B, Gao S, Yu H, He X *et al*. Tumor-associated macrophage-derived exosomes transmitting miR-193a-5p promote the progression of renal cell carcinoma via TIMP2-dependent vasculogenic mimicry. *Cell death & disease* 2022; 13: 382.

2 Wu P, Geng B, Chen Q, Zhao E, Liu J, Sun C *et al*. Tumor Cell-Derived TGFβ1 Attenuates Antitumor Immune Activity of T Cells via Regulation of PD-1 mRNA. *Cancer immunology research* 2020; 8: 1470-1484.

3 Wu P, Cai J, Chen Q, Han B, Meng X, Li Y *et al*. Lnc-TALC promotes O(6)-methylguanine-DNA methyltransferase expression via regulating the c-Met pathway by competitively binding with miR-20b-3p. *Nature communications* 2019; 10: 2045.
