# Supplementary figures and images for: Downregulation of UBB potentiates SP1/VEGFA-dependent angiogenesis in clear cell renal cell carcinoma
Source: Oncogene. 2024 Mar 11;43(18):1386–96. doi: 10.1038/s41388-024-03003-6 (PMC11065696; doi:10.1038/s41388-024-03003-6)

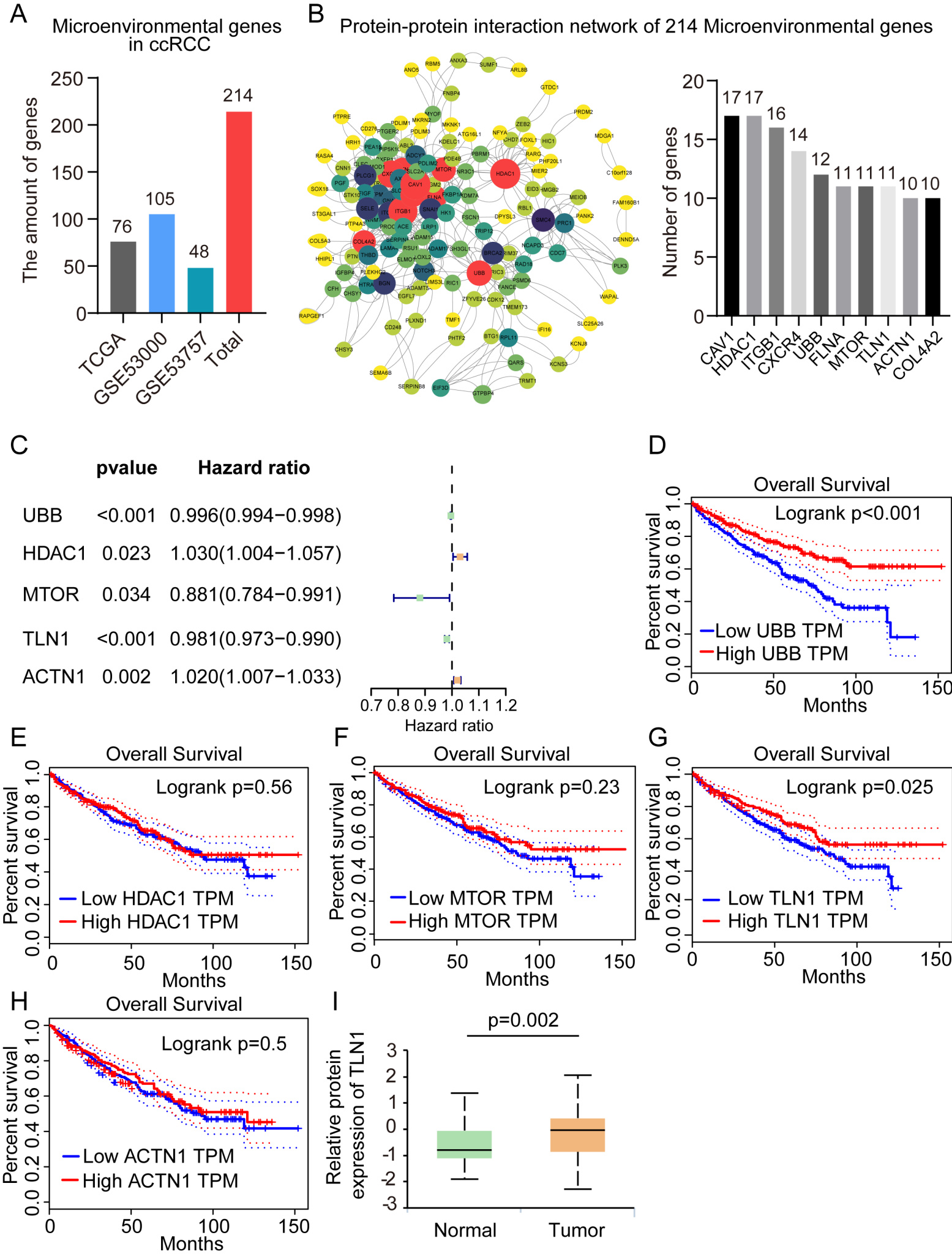

Supplement: Supplementary file 3 — Supplementary Figure 1 [file 41388_2024_3003_MOESM3_ESM.pdf]

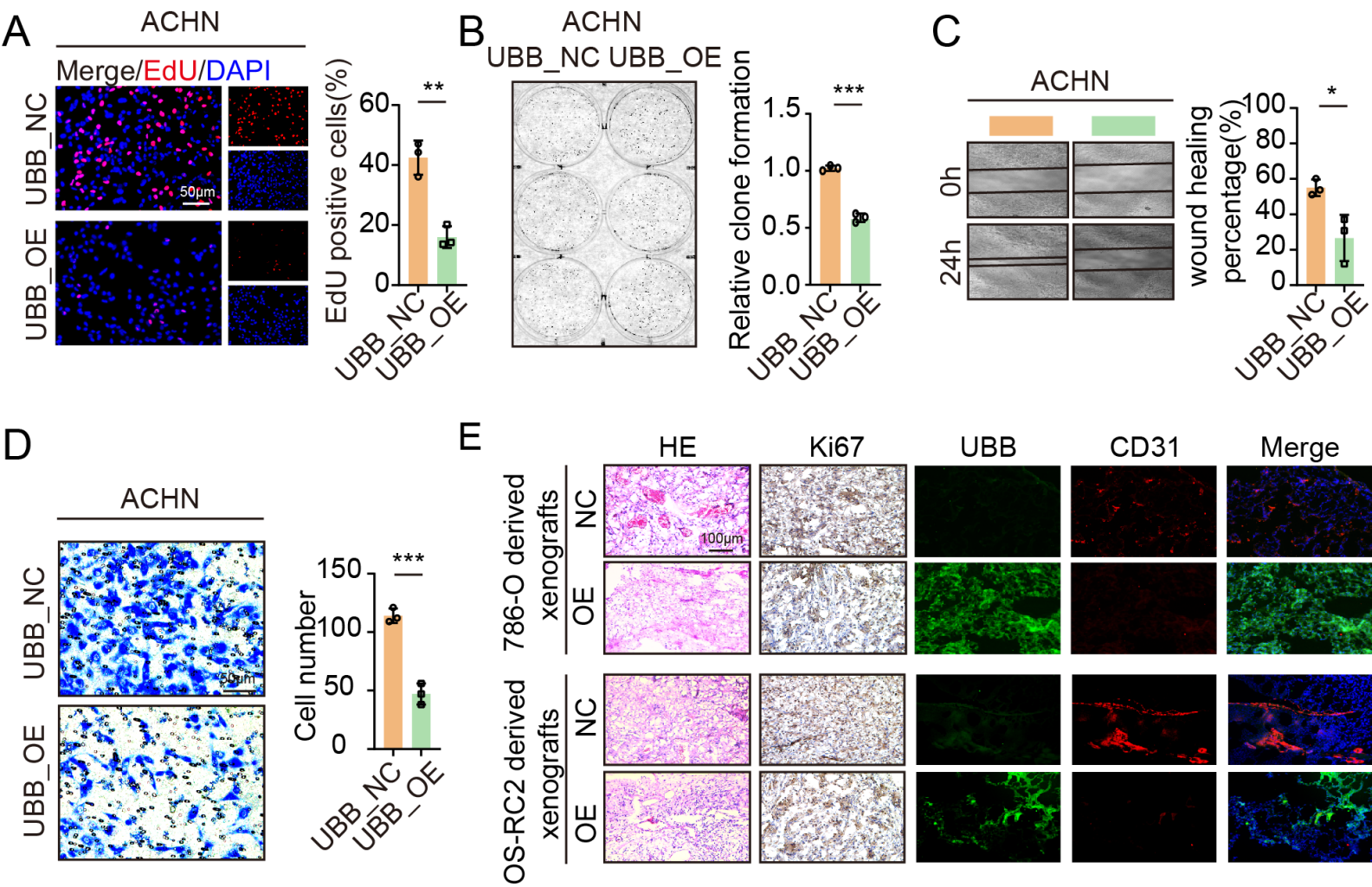

Supplement: Supplementary file 4 — Supplementary Figure 2 [file 41388_2024_3003_MOESM4_ESM.pdf]

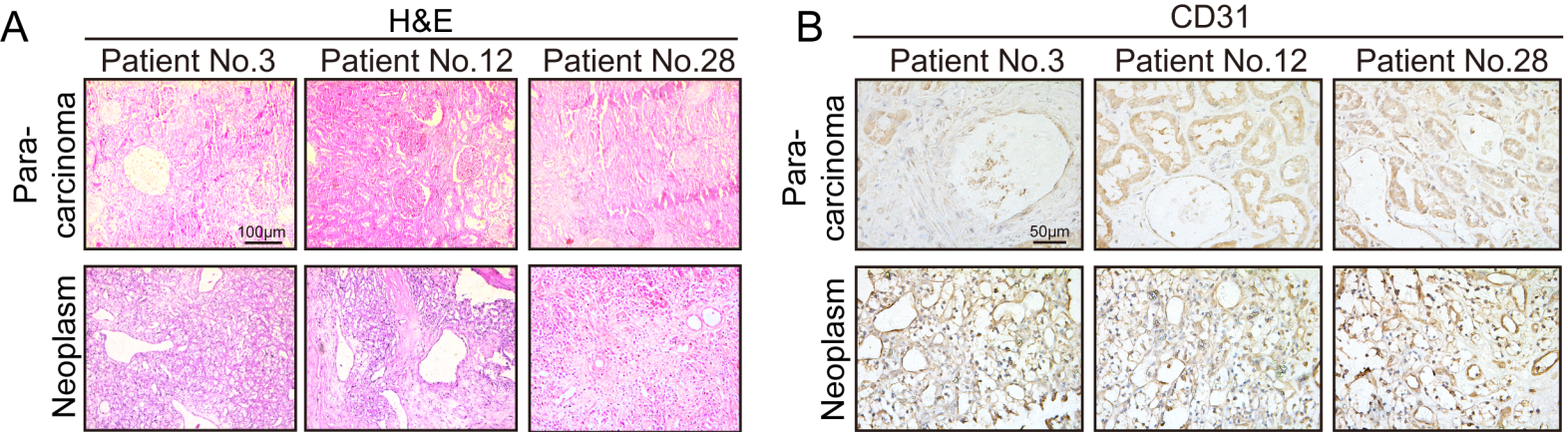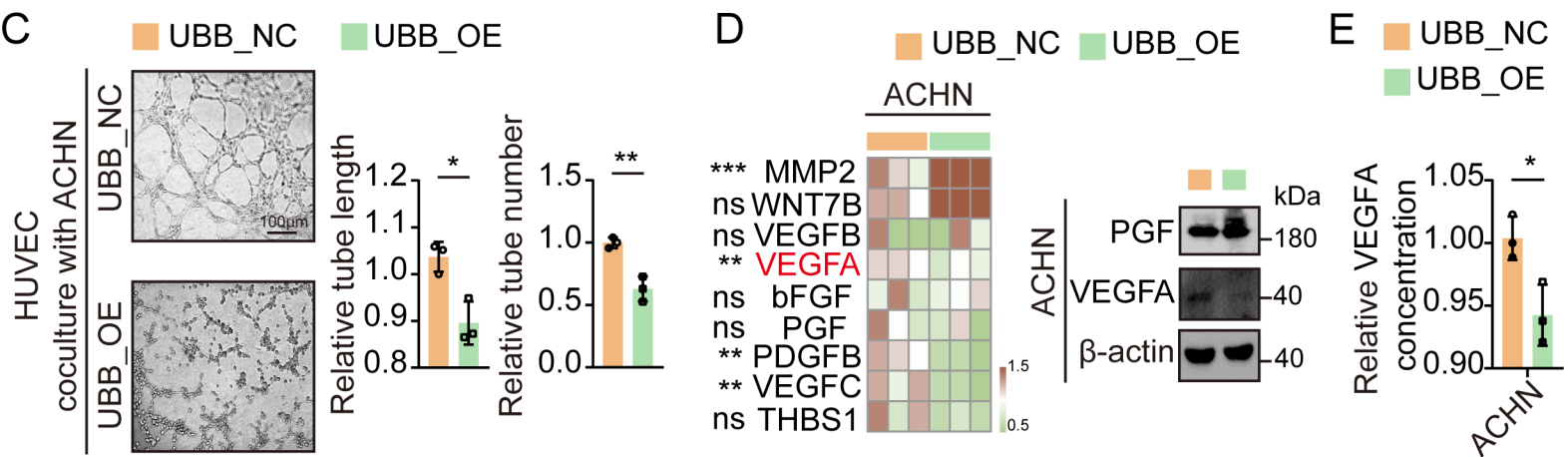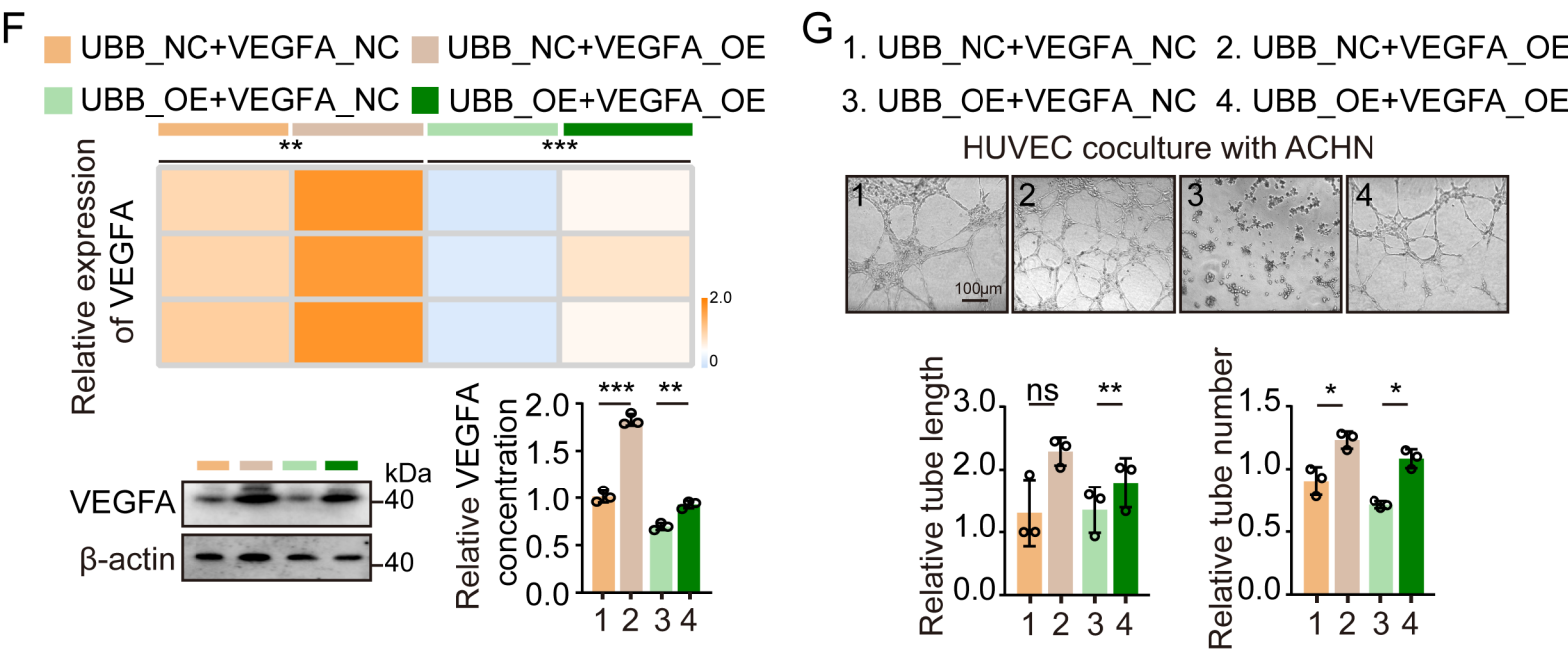

Supplement: Supplementary file 5 — Supplementary Figure 3 [file 41388_2024_3003_MOESM5_ESM.pdf]

# A

## Effect of VEGFA expression level & tumor grade on UALCAN-ccRCC patient survival

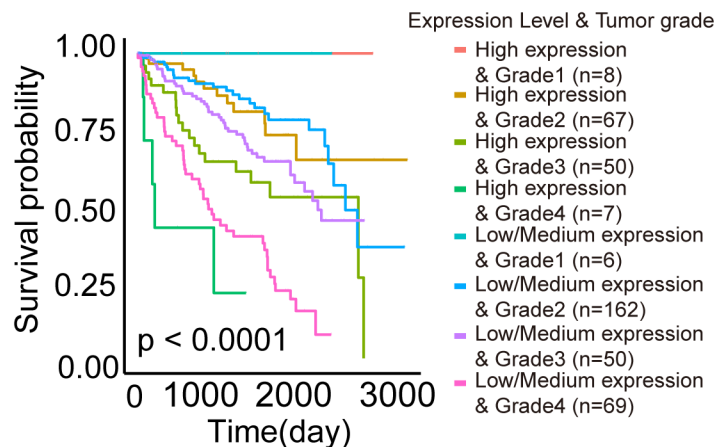

# B

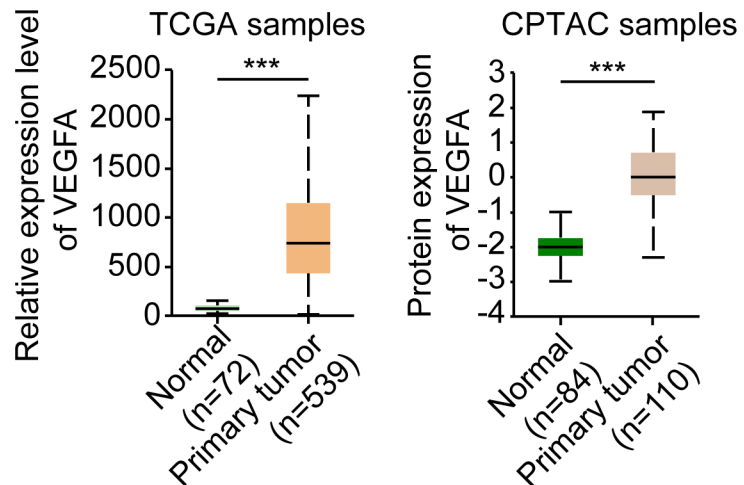

# C

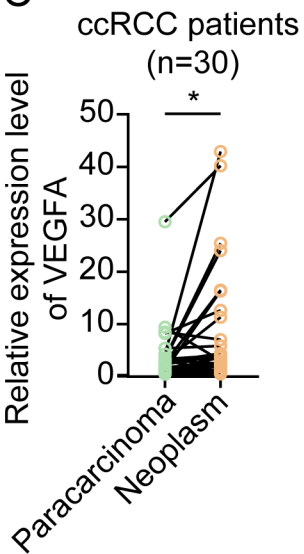

# D

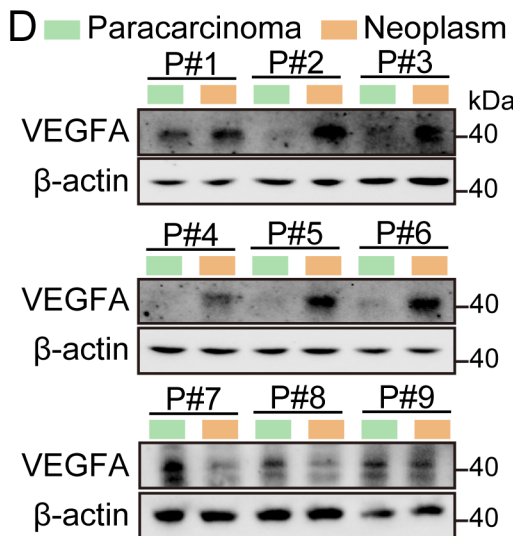

# E

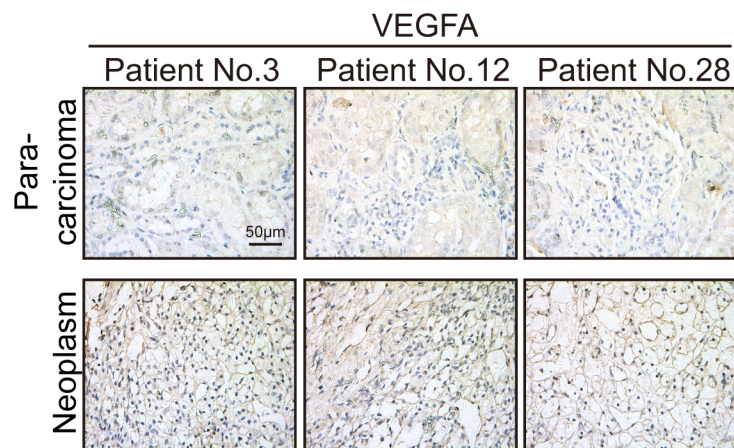

Supplement: Supplementary file 6 — Supplementary Figure 4 [file 41388_2024_3003_MOESM6_ESM.pdf]

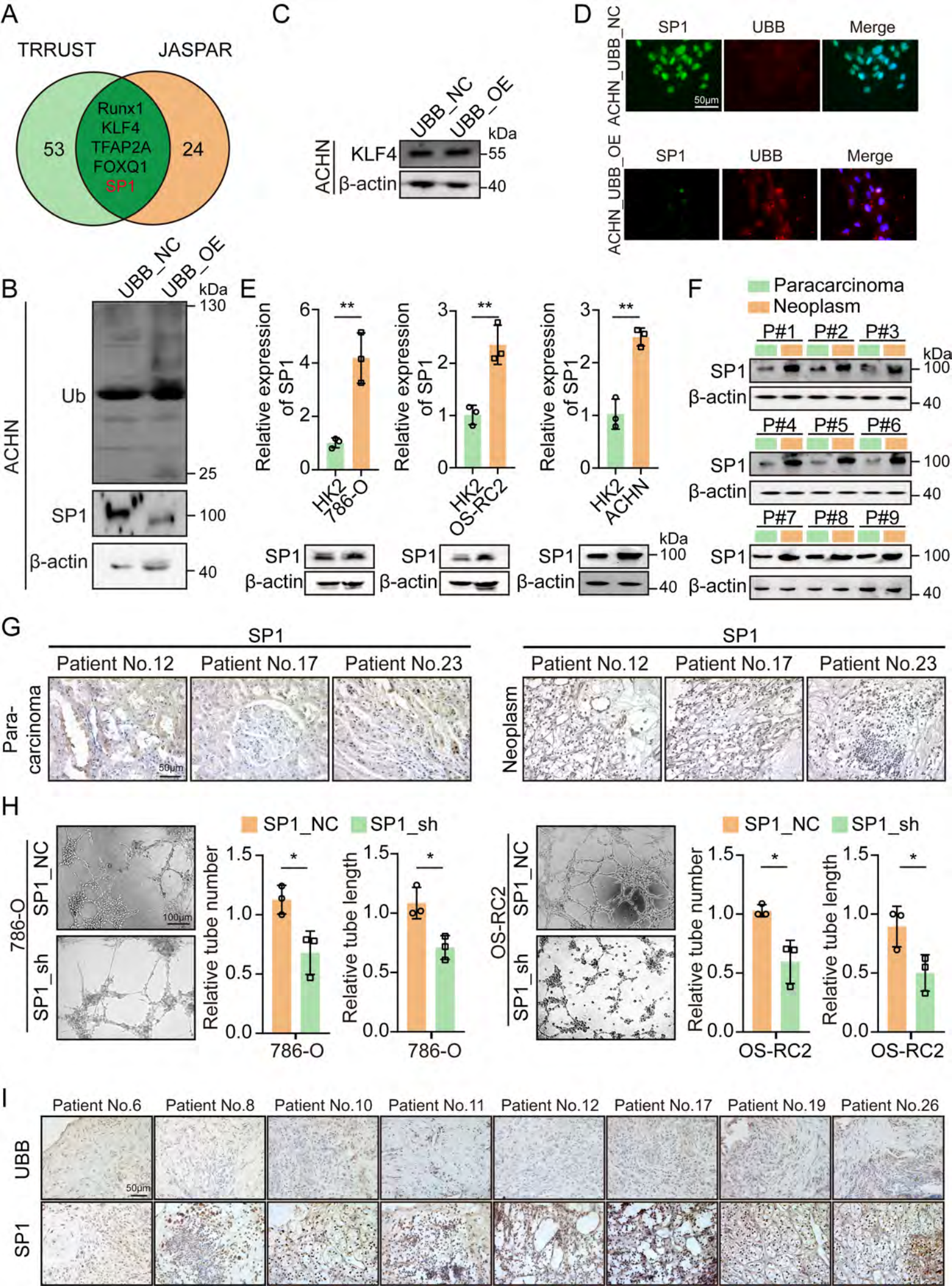

Supplement: Supplementary file 7 — Supplementary Figure 5 [file 41388_2024_3003_MOESM7_ESM.pdf]

**A**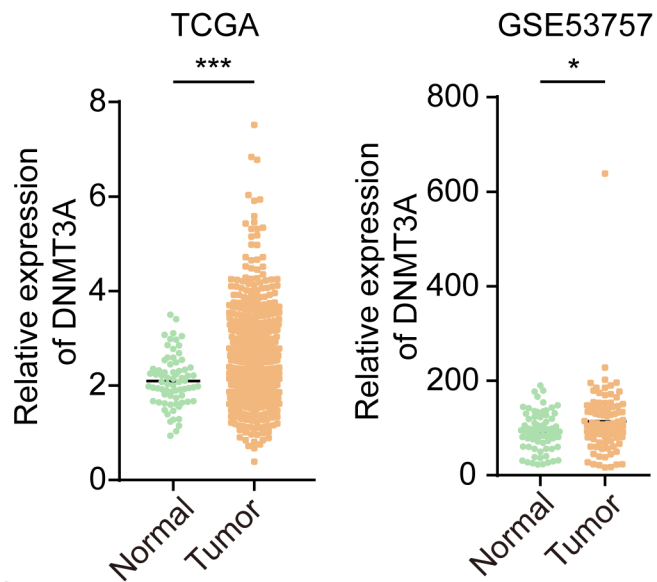**B**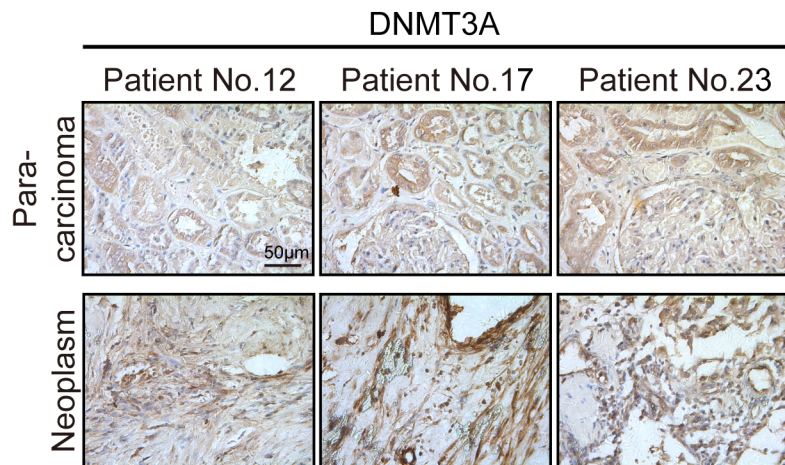**C**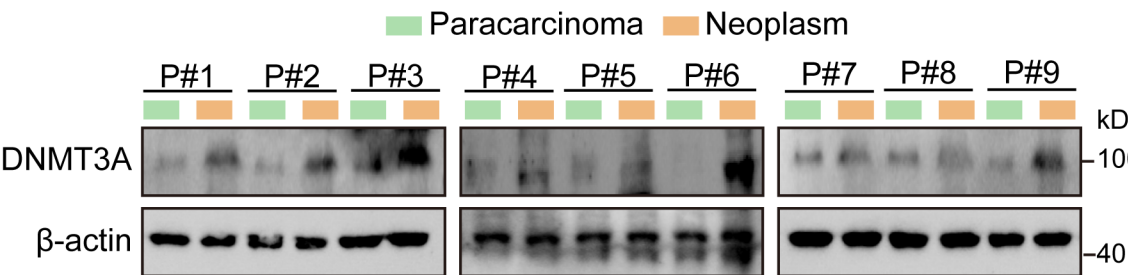**D**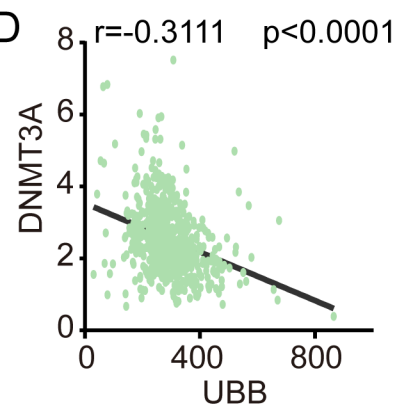

Supplement: Supplementary file 8 — Supplementary Figure 6 [file 41388_2024_3003_MOESM8_ESM.pdf]
