# Supplementary material for: Downregulation of UBB potentiates SP1/VEGFA-dependent angiogenesis in clear cell renal cell carcinoma
Source: Oncogene. 2024 Mar 11;43(18):1386–96. doi: 10.1038/s41388-024-03003-6 (PMC11065696; doi:10.1038/s41388-024-03003-6)
Supplement: Supplementary file 9 — Supplementary table 1 [file 41388_2024_3003_MOESM9_ESM.pdf]

Supplementary Table 1. Patients information

id	futime	fustat	futime (year)
1930642	1324	dead	3.630136986
1942291	177	dead	0.484931507
2196065	362	dead	0.991780822
1848887	1818	alive	4.980821918
2184204	604	dead	1.654794521
2304781	832	alive	2.279452055
2456076	844	alive	2.315068493
2000088	852	dead	2.336986301
2291727	854	alive	2.342465753
2276840	878	alive	2.408219178
1856379	1036	dead	2.84109589
1895433	1185	dead	3.249315068
1868704	1231	dead	3.37260274
1938443	1318	dead	3.61369863
2107232	1372	dead	3.75890411
2317042	1424	dead	3.904109589
1931637	1741	alive	4.769863014
1927811	1752	alive	4.802739726
1927811	1752	alive	4.802739726
1823327	1757	dead	4.816438356
1914167	1765	alive	4.838356164
1905715	1795	alive	4.920547945
1907820	1799	alive	4.931506849
1902513	1807	alive	4.950684932
1895433	511	dead	1.4
1891533	1820	alive	4.98630137
1891533	1820	alive	4.98630137
1898347	1821	alive	4.989041096
1890715	1837	alive	5.035616438
1944047	1701	dead	4.660273973
