# Supplementary material for: Downregulation of UBB potentiates SP1/VEGFA-dependent angiogenesis in clear cell renal cell carcinoma
Source: Oncogene. 2024 Mar 11;43(18):1386–96. doi: 10.1038/s41388-024-03003-6 (PMC11065696; doi:10.1038/s41388-024-03003-6)
Supplement: Supplementary file 10 — Supplementary Table 2 [file 41388_2024_3003_MOESM10_ESM.pdf]

Supplementary Table 2. Antibodies used

Antibody	Company	catalog	Application
β-actin	Proteintech	81115-1-RI	1:5000 for WB
UBB	Proteintech	10201-2-Al	1:1000 for WB, 1:100 for IHC, 1:50 for IF, 1:50 for IP
SP1	Proteintech	66508-1-Ig	1:1000 for WB, 1:100 for IHC, 1:50 for IF, 1:50 for IP
DNMT3A	Cell Signal	32578	1:1000 for WB, 1:100 for IHC, 1:50 for IP, 1:50 for ChIP
DNMT3A	Abcam	ab237985	1:50 for IF
PGF	Cell Signal	3174	1:1000 for WB
H3K23me3	Abcam	ab6002	1:50 for ChIP
IgG	Santa Cruz	sc-2027	1:50 for IP
Flag	Sigma-Aldı	M8823	1:50 for IP
Myc	Cell Signal	2276	1:50 for IP
VEGFA	Proteintech	19003-1-Al	1:1000 for WB, 1:100 for IHC, 1:50 for IF
CD31	Proteintech	66065-2-Ig	1:100 for IHC, 1:50 for IF
