# Supplementary material for: Downregulation of UBB potentiates SP1/VEGFA-dependent angiogenesis in clear cell renal cell carcinoma
Source: Oncogene. 2024 Mar 11;43(18):1386–96. doi: 10.1038/s41388-024-03003-6 (PMC11065696; doi:10.1038/s41388-024-03003-6)
Supplement: Supplementary file 11 — Supplementary Table 3 [file 41388_2024_3003_MOESM11_ESM.pdf]

Supplementary Table 3. Primer used

Primer Name	Primer Sequence(5’-3’）
hsa-UBB-Forward	GGTGAGCTTGTTTGTGTCCCTGT
hsa-UBB-Reverse	TCCACCTCAAGGGTGATGGTC
hsa-VEGFA-Forward	AGGGCAGAATCATCACGAAGT
hsa-VEGFA-Reverse	AGGGTCTCGATTGGATGGCA
hsa-VEGFB-Forward	GAGATGTCCCTGGAAGAACACA
hsa-VEGFB-Reverse	GAGTGGGATGGGTGATGTCAG
hsa-VEGFC-Forward	ATGTGTGTCCGTCTACAGATGT
hsa-VEGFC-Reverse	GGAAGTGTGATTGGCAAAACTGA
hsa-PGF-Forward	GAACGGCTCGTCAGAGGTG
hsa-PGF-Reverse	ACAGTGCAGATTCTCATCGCC
hsa-bFGF-Forward	AGAAGAGCGACCCTCACATCA
hsa-bFGF-Reverse	CGGTTAGCACACACTCCTTTG
hsa-PDGFB-Forward	CTCGATCCGCTCCTTTGATGA
hsa-PDGFB-Reverse	CGTTGGTGCGGTCTATGAG
hsa-WNT7B-Forward	CACAGAAACTTTCGCAAGTGG
hsa-WNT7B-Reverse	GTACTGGCACTCGTTGATGC
hsa-MMP2-Forward	TACAGGATCATTGGCTACACACC
hsa-MMP2-Reverse	GGTCACATCGCTCCAGACT
hsa-THBS1-Forward	AGACTCCGCATCGCAAAGG
hsa-THBS1-Reverse	TCACCACGTTGTTGTCAAGGG
hsa-SP1-Forward	GAGAAAACAGCCCAGATGC
hsa-SP1-Reverse	CCCTTCCTTCACTGTCTTT
