# Supplementary material for: Downregulation of UBB potentiates SP1/VEGFA-dependent angiogenesis in clear cell renal cell carcinoma
Source: Oncogene. 2024 Mar 11;43(18):1386–96. doi: 10.1038/s41388-024-03003-6 (PMC11065696; doi:10.1038/s41388-024-03003-6)
Supplement: Supplementary file 12 — Supplementary Table 4 [file 41388_2024_3003_MOESM12_ESM.pdf]

Supplementary Table 4. ChIP Primer used

Primer Nan	Primer Sequence(5’-3’）
UBB_prim	GGCCAGGCTGGTCTTGAACC
UBB_prim	TTTCACGACCCTAATGTCCGC
